# Cryo-ablation management of atrial fibrillation in Fabry disease without agalsidase alpha: a case report

**DOI:** 10.3389/fcvm.2025.1483283

**Published:** 2025-05-30

**Authors:** Yuanzhu Li, Bi Huang, Suxin Luo

**Affiliations:** ^1^ Department of Cardiology, The First Affiliated Hospital of Chongqing Medical University, Chongqing, China; ^2^ Cardiovascular Disease Laboratory, Chongqing Medical University, Chongqing, China

**Keywords:** Fabry disease (FD), paroxysmal atrial fibrillation, cryo-ablation, ventricular hypertropy, molecular docking

## Abstract

Fabry disease (FD) is a rare genetic disorder caused by mutations in the *GLA* gene, affecting multiple organs. Over 60% of patients experience heart-related issues, primarily arrhythmias. Unlike typical cases, these arrhythmias are complex and often do not respond well to standard antiarrhythmic drugs, sometimes worsening symptoms. This paper presents a case study of a FD patient with paroxysmal atrial fibrillation successfully treated using bilateral pulmonary vein cryoablation, resulting in positive outcomes. Additionally, we conducted molecular docking studies for the first time to assess the enzyme-substrate binding of E358del, confirming findings similar to laboratory experiments. Our findings underscore the potential role of artificial intelligence in better understanding FD, and aim to provide insights into managing arrhythmias associated with FD.

## 1 Introduction

Fabry disease (FD) is a rare genetic disorder due to mutations in the galactosidase alpha (GLA) gene, leading to partial or complete loss of activity of the encoded α-galactosidase A (α-Gal A). This deficiency inhibits the breakdown of globotriaosylceramide (Gb3), resulting in its accumulation in lysosomes and causing a multiple-system disease (1). Clinically, the presentations of FD patients vary significantly, from asymptotic to classic form, but generally, female patients tend to have less typical presentation compared with male patients (2).

Among the affected systems, more than 60% of FD patients display symptoms of cardiac involvement, which is also the primary cause of death for such patients. FD patients often present with left ventricular hypertrophy, arrhythmia, and heart valvular disease (3–5). Notably, arrhythmias induced by FD exhibit diversity, with commonly prescribed antiarrhythmic medications showing limited effectiveness and even worsening their symptoms (5, 6).

Presently, more than 900 types of *GLA* gene mutations have been identified as pathogenic or likely pathogenic. Different gene mutation sites determine distinct residual enzyme activities, thereby impacting the age of onset and the progression in individuals with FD (7, 8). Here we report a female FD patient with delayed onset, primarily presented with atrial fibrillation (AF), and rhythm control was used successfully with cryo-ablation. Through SWISS-MODEL analysis, the enzyme protein structure in this patient was compared with wild-type control. This case contributes to the clinical understanding of the c.1072_1074delGAG (p.E358del) mutation, offering insights for treating FD patients presenting with arrhythmia complications.

## 2 Case presentation

### 2.1 Clinical presentation and physical examination

**Figure 1:**
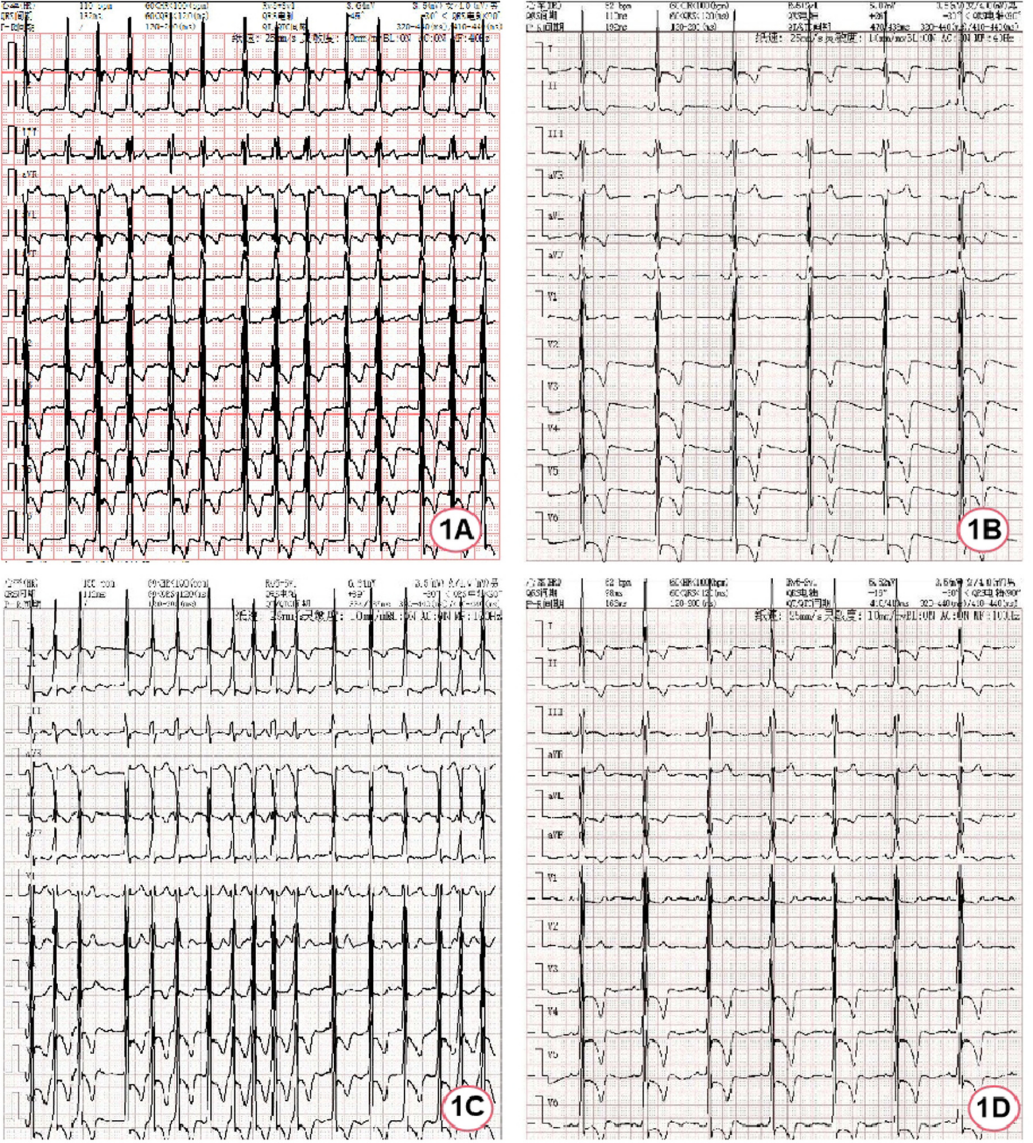
ECG of the patient during hospitalization. **(A)** ECG at admission; **(B)** ECG after spontaneous restoration of sinus rhythm; **(C)** ECG during atrial fibrillation episodes; **(D)** ECG after bilateral pulmonary vein cryoablation.

A 50-year-old female patient was admitted due to recurrent chest pain. During the past six years, the patient experienced recurrent episodes of unexplained dull pain in the precordial region, exacerbated by physical activity. Throughout the clinical course, the patient exhibited no signs of lower extremity edema, paroxysmal nocturnal dyspnea, or systemic manifestations such as hypohidrosis, oliguria, acral pain, gastrointestinal disturbances, or visual impairment. Furthermore, there was no evidence of stroke-related symptoms, including transient ischemic attacks, focal neurological deficits, or other cerebrovascular events. Comprehensive clinical evaluations revealed no abnormalities in renal function or proteinuria. The day before admission, the patient felt palpitation and meanwhile, the symptom of chest pain worsened with sweating. An emergency electrocardiogram (ECG) indicated atrial flutter (Figure 1A), prompting her admission to the cardiovascular department for further assessment and treatment of the arrhythmia.

Four years ago, the patient was diagnosed with hypertrophic cardiomyopathy and echocardiography revealed symmetric left ventricular hypertrophy (14 mm). Electrocardiography showed sinus bradycardia, frequent atrial premature beats, and paroxysmal atrial tachycardia. She denied the history of hypertension, diabetes mellitus, or coronary atherosclerotic heart disease. Her mother passed away 20 years ago due to “heart disease”. She also denied the presence of cardiovascular diseases in other family members.

Upon admission, her vital signs were stable with blood pressure of 113/76 mmHg (1 mmHg = 0.133 kPa). Physical examination showed no rash or other skin lesions were observed on the patient's body. Hearing and vision were normal, with no abnormalities detected on eye examination. Pulmonary auscultation revealed no rales. The cardiac borders were not enlarged; heart rate was 87 beats per minute, with a regular rhythm and no murmurs detected. No edema was noted in the lower extremities. Neurological examination revealed no positive signs.

### 2.2 Assistant examination

After hospitalization, myocardial enzymes revealed elevated levels of troponin I at 0.27 ng/ml (normal range 0–0.023 ng/ml) and N-terminal pro-B-type natriuretic peptide were 3,910 ng/L (normal range 0–300 ng/L). The first electrocardiogram after hospitalization was completed 40 min after the emergency electrocardiogram and it had restored to sinus rhythm without using antiarrhythmic drugs, but it was remarkable sinus bradycardia (47 bpm) (Figure 1B). Afterwards, recurrent episodes of palpitation occurred and ECG indicated rapid ventricular response AF (Figure 1C). Due to the obvious symptoms, rhythm control strategy was used with intravenous use of amiodarone. Meanwhile, anticoagulation with dabigatran etexilate was administered. Nineteen hours later, it successfully restored to sinus rhythm, showing sinus bradycardia (52 bpm).

**Figure 2:**
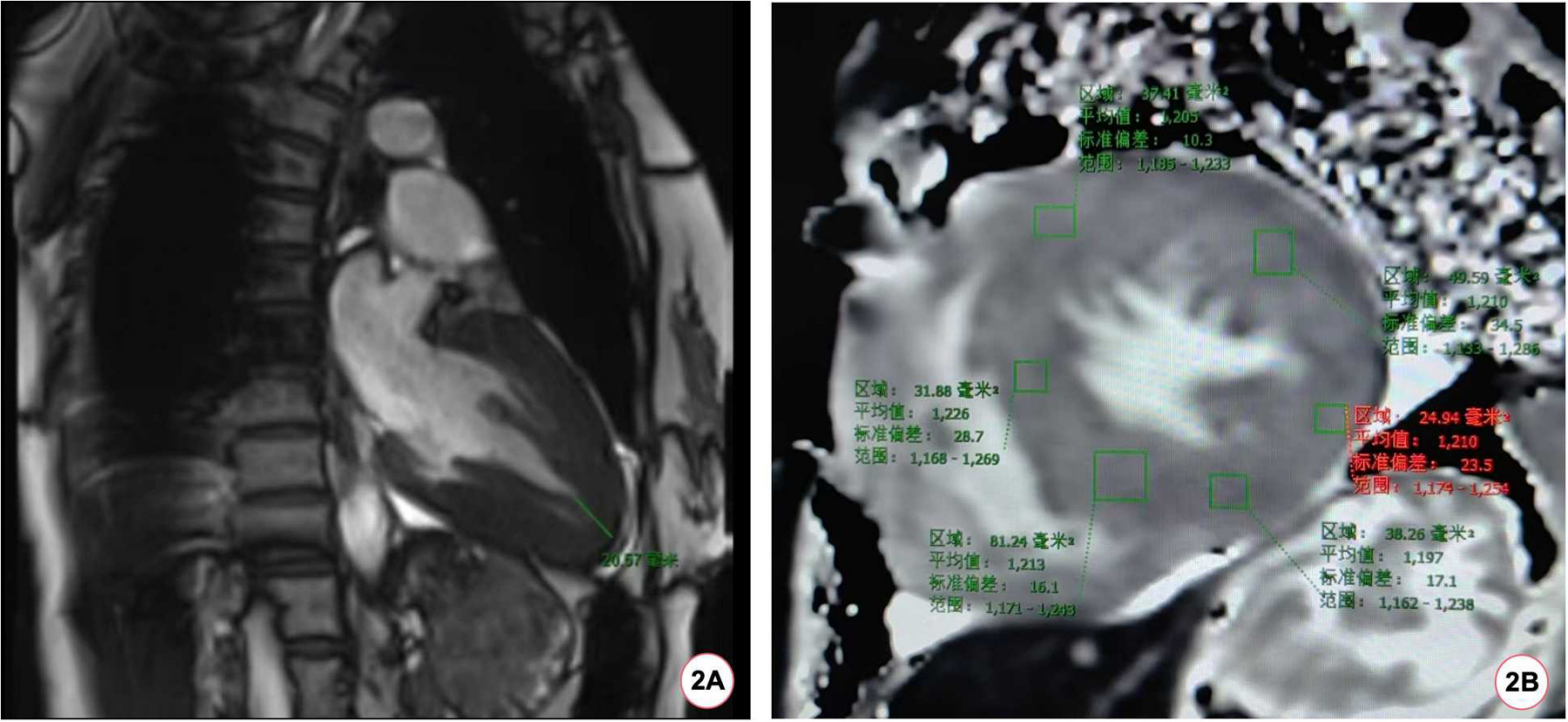
The patient's CMR scan. **(A)** The patient's CMR imaging revealed a ventricular wall thickness exceeding 20 mm at the apex of the heart. **(B)** The baseline myocardial T1 mapping value of the patient's CMR was 1,210 (±23.5) ms.

During the hospitalization, Holter displayed an average ventricular rate of 53 bpm and echocardiography showed a left ventricular ejection fraction of 65%, interventricular septal end-diastolic thickness of 15 mm, left ventricular posterior wall end-diastolic thickness of 15 mm, maximum thickness at apex 18 mm without left ventricular outflow obstruction. Cardiac magnetic resonance (CMR) imaging also revealed diffuse hypertrophy in the apical and mid-ventricular walls of the left ventricle, approximately 21 mm in the apical (Figure 2A) and the T1 mapping value was 1,210 ± 23.5 ms (Figure 2B). Ambulatory blood pressure test showed the average 24-hour blood pressure was 120/72 mmHg. Coronary angiography did not show obvious stenosis or slow flow of coronary artery. Considering her recurrent episodes of paroxysmal AF/atrial flutter with obvious symptoms, the patient received bilateral pulmonary vein cryo-ablation, after which it reverted to sinus rhythm (Figure 1D).

### 2.3 Etiological results

Due to the patient's unexplained left ventricular hypertrophy, along with arrhythmias, and suspicious cardiac family history, disease associated with gene mutation was suspected. Subsequent examination showed her α-Gal A activity was decreased and globotriaosylsphingosine (Lyso-GL-3) concentration was increased (Table 1). Finally, whole-genome sequencing identified a heterozygous mutation at c.1072_1074delGAG (p.E358del) in the patient's *GLA* gene (Table 2).

**Table 1:** Patient's peripheral blood α-Gal A enzyme activity and globotriaosylsphingosine (Lyso-GL-3) concentration.

Methodology: MSMS				
Test item	Abbr.	Test result	Reference interval	Unit
Alpha-galactosidase A	α-Gal A	1.09 ↓	2.40–17.65	umol/L/h
Biomarkers(Lyso-GL-3)	Lyso-GL-3	8.61 ↑	<1.11	ng/ml

**Table 2:** Whole gene sequencing results of the patient.

Genes and transcripts	Related genes and genetic patterns	Chromosome position	Variable site
GLA NM_000169.2	Fabry disease (XL)	chrX:10065301 3-100653015	c.1072_1074 delGAG p.E358del
Exon/Intron	Heterozygosity	gnomAD maximum frequency	Mutation degree
exon7	heterozygote	-	likely pathogenic

**Figure 3:**
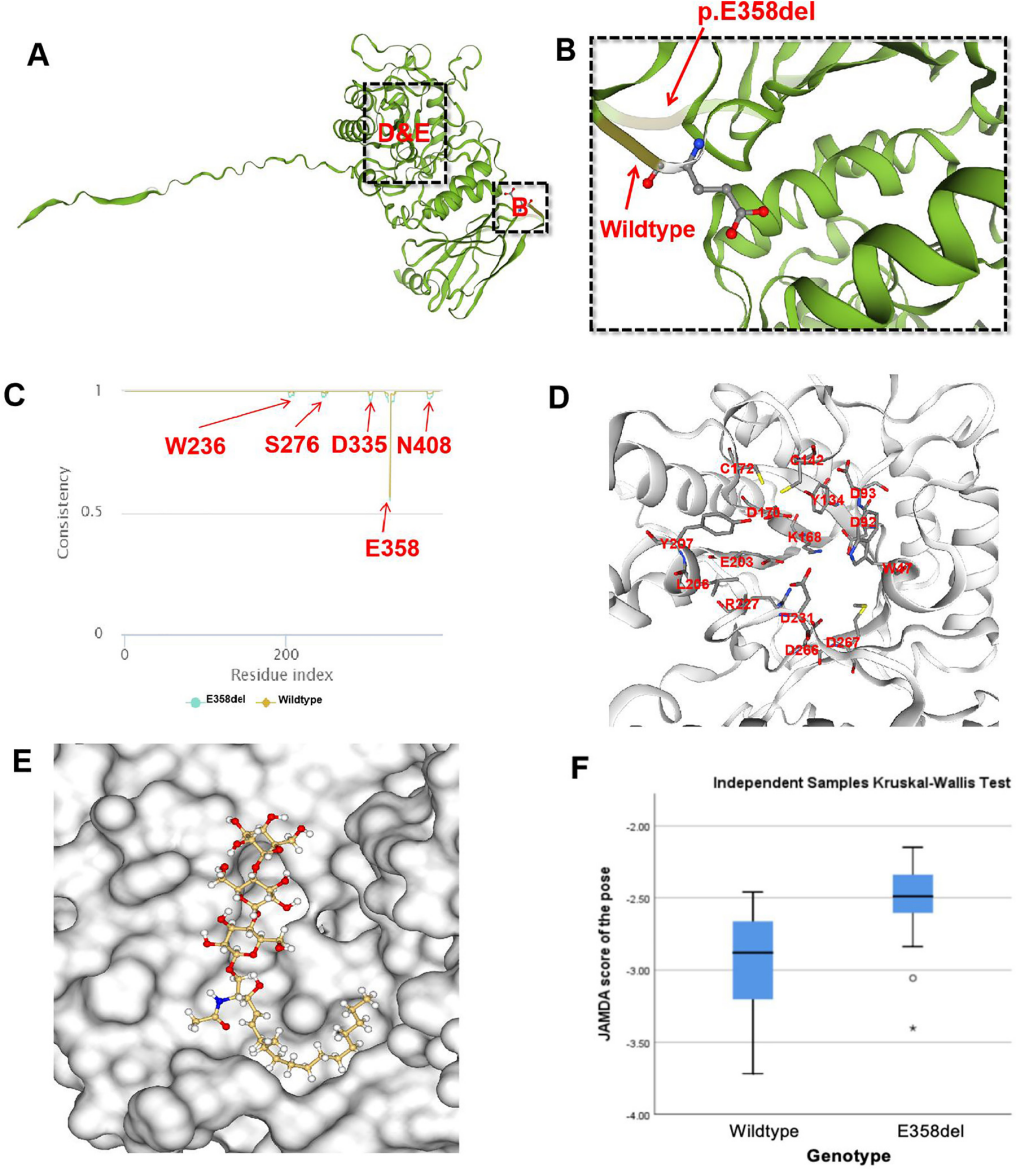
Molecular docking of E358del mutation. **(A,B)** SWISS-MODEL simulation comparing wild-type and p.E358del; **(C)** consistency analysis of α-Gal A encoded by wild-type and E358del mutations; **(D)** One of the molecular pockets in α-Gal A dimer; **(E)** schematic diagram of Gb3 binding to α-Gal A in E358del mutant (JAMDA score = −3.4018); **(F)** non-parametric test results of JAMDA scores for wild-type and E358del mutant docking with Gb3.

The MutationTaster tool demonstrated this mutation was pathogenic (prob: 0.999) (9). The American College of Medical Genetics and Genomics also classified this mutation as likely pathogenic. Finally, we used SWISS-MODEL to visually simulate the structural site (Figures 3A,B) of E358del mutation (10–14). As α-Gal A is sheared and assembled into dimers *in vivo*, we utilize the dimeric form of α-Gal A for subsequent experiments. Then we performed a consistency analysis using wild-type and mutant (E358del) dimer structures. We observed significant disparities not only in amino acid 358 but also in the vicinity of amino acids 236, 278, 335, and 405 (Figure 3D). To assess the docking capability of α-Gal A with substrates, we employed the JAMDA Protein-ligand docking function in ProteinsPlus for docking (15–19). Initially, we retrieved the molecular coordinate file of globotriaosylceramide (Gb3, Compound CID: 66616222) from the PubChem website and identified pockets formed by W47, D92, D93, Y134, C142, K168, D170, C172, E203, L206, Y207, R227, D231, D266, and M267 amino acids for docking (20) (Figure 3E). The wild-type yielded 29 poses and JAMDA scores, whereas the E358del variant produced 32 poses and JAMDA scores (19). Finally, we validated the significant difference between wild-type and E358del using non-parametric tests using the SPSS statistical software, version 26.0 (IBM, USA) (Figure 3F), which suggested that the E358del mutant exhibits significantly diminished substrate binding ability compared to that of the wild-type.

### 2.4 Follow-up

After cryo-ablation for AF, sinus rhythm was maintained. The patient was advised to receive enzyme replacement therapy (ERT), but she refused. Over a 2-year follow-up, the patient had no symptoms of discomfort and ECG confirmed the maintenance of sinus rhythm (Figure 1D).

## 3 Discussion

FD is a rare lysosomal storage disorder caused by mutations in the *GLA* gene on the X chromosome. Epidemiological study shows that more than 60% of patients with FD have cardiac involvement, among which left ventricular hypertrophy is common (3). Therefore, the phenotype of left ventricular hypertrophy is usually an important clue to proceed the subsequent examination to exclude FD.

In clinical practice, the common cause of left ventricular hypertrophy includes hypertension, aortic valve stenosis, hypertrophic cardiomyopathy, amyloidosis, FD, glycogen storage syndrome, etc (21). In this patient, hypertension and aortic valve stenosis were excluded based on the medical history, ambulatory blood pressure monitoring, and echocardiography. In such a case, hypertrophic cardiomyopathy is usually considered. However, hypertrophic cardiomyopathy mainly characterized by asymmetric hypertrophy of the interventricular septum mostly caused by mutations in the sarcomere gene (22), whereas this patient's left ventricular hypertrophy was symmetrical. Another characteristic of FD is the involvement of the cardiac conduction system, leading to sinus bradycardia or bradycardia-tachycardia syndrome caused by sinus node involvement in the early stages of the disease (23, 24).

AF is the most common arrhythmia with an incidence of nearly 20% in FD (4, 5). Notably, arrhythmias in patients with FD have multiplicity and complexity. In this case, the patient mainly presented with paroxysmal atrial flutter and AF, but the heart rate was slow during sinus rhythm, making the use of antiarrhythmic drugs cautious (25). In addition, literature reports suggest that amiodarone can inhibit lysosomal degradation and induce phospholipid deposition in organs, which may exacerbate the symptoms of FD (6). Therefore, rhythm control with antiarrhythmic drugs for such patients were challenging and ablation is an alternative treatment. Up to now, there have been very scarce reports on the use of ablation for AF in patients with FD. Qian et al (25). reported a case with FD complicated with AF and successfully treated with ablation, but in this study, a traditional radiofrequency ablation was adopted and underwent repeated ablation. In contrast, we used cryo-ablation to treat AF in our case. Although previous studies did not demonstrated the superiority of cryo-ablation to traditional radiofrequency ablation (26), whether cryo-ablation is superior to radiofrequency ablation in patients with FD deserves further study.

Currently, ERT is the main treatment for FD via intravenous administration of exogenous agalsidase alpha/beta every two weeks to replace the function of deficient endogenous α-Gal A (27). ERT has demonstrated efficacy in enhancing the quality of life and mitigating adverse events among FD patients (28). However, ERT treatment has several limitations. For instance, most exogenous enzymes are primarily taken up by the liver, resulting in limited amounts reaching the heart and kidneys (27). Furthermore, postmortem analyses have demonstrated some Gb3 accumulation in the brains in FD patients; however, no exogenous enzyme therapy currently available has been shown to traverse the blood-brain barrier although the clinical significance of Gb3 accumulation in the brain remains unclear (27). Moreover, the autopsy results indicated that FD patients had extensive accumulation of Gb3 in their brains, but currently, there is no exogenous enzyme capable of crossing the blood-brain barrier (27). In addition, approximately 40% of male patients undergoing ERT may develop drug antibodies, which may neutralize the drug's efficacy (28). In this context, novel treatment strategies are being explored. Missense mutations in the *GLA gene* have been shown to have normal or slightly decreased enzyme activity, but α-Gal A cannot enter lysosome and is degraded by endoplasmic reticulum due to structural abnormalities (29). These mutations are now considered to be more effective through the addition of an oral chaperone therapy regimen (30). In comparison to ERT, oral chaperone therapy does not stimulate antibody production and maintains enzyme levels more closely to physiological states over an extended period (28, 31). In our molecular docking experiments, we demonstrated a notable decrease in docking efficacy with substrates following the c.1072_1074 delGAG mutation. The consistency analysis between the wild-type and E358del mutant revealed variances in the protein structure surrounding W236, S276, D335, E358, and N408 (Figure 3E). This observation aligns with the findings of Seiji et al. (32), who illustrated the significant role of hydrogen bonding between E358 and W236 in maintaining the α-Gal A conformation. However, the roles other amino acids played in E358del mutations require further experimental verification.

Changes in critical sites of enzyme structure significantly influence molecular docking capability (Figure 3H), which aligns with previous reports indicating the limited effectiveness of chaperone therapy for E358del mutation (33). Moreover, some *GLA* gene mutations are related to specific clinical phenotypes, for instance, mutation at N215S or R112H is commonly referred as “cardiac mutations” (34). Multiple studies targeting heterozygous female FD patients have shown that up to 60%–100% of patients have subjective and/or objective evidence of FD, with approximately 10% exhibiting severe symptoms, suggesting that simply X-linked single-gene inheritance patterns cannot explain the complex phenotype in FD (35).

We searched for c.1072_1074delGAG mutations in the *GLA* gene in the ClinVar database, and found a total of 9 patients with clinical symptoms and phenotypes reported in 6 articles (36–41). The characteristics of all patients associated with this gene mutation, including the one reported in our study, are summarized in Table 3. Among the affected organs, the heart, kidneys, and nervous system were each involved in 5 cases, followed by the cornea, which was involved in 4 cases.

**Table 3:** Summary of clinical manifestations of all NM:000169.3 (*GLA*): c.1072_1074del mutations in the ClinVar database.

Authors	Lianne C (36)	Wu (37)			Markus (38)	Lorenzo (39)		Takaaki (40)	Kenichi (41)	Our present case
Clinical phenotype	Classic	Classic	Classic	Non-classic	Classic			Classic	Classic	Non-classic
Age (years)	In thirties	25	22		6	50		4	8	50
Gender	Male	Male	Male	Female	Male	Male	Female	Male	Male	Female
Race	Australian	Chinese	Chinese	Chinese	Caucasian			Japanese	Japanese	Chinese
Skin lesions	+	+	+	–	–					–
Neurosensory abnormalities		+	+	–	+			+	+	–
Cardiovascular involvements		+	–	–	–	+	+		+	+
Renal insufficiency	+	+	+	–	–	+			+	–
Hypohidrosis		–	+	–	–			+	+	–
Enteropathy		–	–	–	+					–
Cornea verticillata		+	+	+	+					
Enzyme activity	0.4 nmol/ml/h	0.9 nmol/h/ml	1.0 nmol/h/ml	2.6 nmol/h/ml	1.4% of WT	12% of WT		4.7 AgalU		1.09 umol/L/h

Despite the patient did not receive ERT treatment, her sinus rhythm remained for more than 2 years following bilateral pulmonary vein cryo-ablation, which indicated, for one hand, cryo-ablation is effective for such patients; for the other hand, cryo-ablation can be served as a temporary symptom-alleviated method even if ERT is not administered. Actually, previous studies have demonstrated the benefit of early rhythm control vs. rate control in patients with AF (42). Although the efficacy of ablation in FD patients is still needed to be confirmed, ablation remains a therapeutic option for FD with symptomatic AF.

## 4 Conclusion

For patients with unexplained cardiac hypertrophy, especially middle-aged women, even if there is a lack of multi-system involvement, genetic test should be administered to exclude FD. Cryo-ablation of bilateral pulmonary veins may be an effective way to treat AF secondary to FD.

## Data Availability

The datasets presented in this study can be found in online repositories. The names of the repository/repositories and accession number(s) can be found in the article/Supplementary Material.
